# From evidence to understanding: a commentary on Fisher (1922) ‘On the mathematical foundations of theoretical statistics’

**DOI:** 10.1098/rsta.2014.0252

**Published:** 2015-04-13

**Authors:** David J. Hand

**Affiliations:** Imperial College London, London, UK

**Keywords:** inference, inverse probability, parameter, statistic, likelihood

## Abstract

The nature of statistics has changed over time. It was originally concerned with descriptive ‘matters of state’—with summarizing population numbers, economic strength and social conditions. But during the course of the twentieth century its aim broadened to include inference—how to use data to shed light on underlying mechanisms, about what might happen in the future, about what would happen if certain actions were taken. Central to this development was Ronald Fisher. Over the course of his life he was responsible for many of the major conceptual advances in statistics. This is particularly illustrated by his 1922 paper, in which he introduced many of the concepts which remain fundamental to our understanding of how to extract meaning from data, right to the present day. It is no exaggeration to say that Fisher's work, as illustrated by the ideas he described and developed in this paper, underlies all modern science, and much more besides. This commentary was written to celebrate the 350th anniversary of the journal *Philosophical Transactions of the Royal Society*.

Ronald Fisher's seminal 1922 paper ‘On the mathematical foundations of theoretical statistics’ [1] was submitted to the Royal Society on 25 June 1921, read on 17 November of that same year, and appeared in the Society's journal on 19 April the following year. A modern-day statistician reading the paper cannot but be amazed at the contribution. At first glance, this single paper appears to introduce a number of concepts which one might have expected to develop gradually over decades, each being introduced in a seminal paper of its own. These include consistency, efficiency, sufficiency, validity, and, of course, likelihood. However, the appearance of creation from nothing is in fact a little misleading. The 1922 paper did not appear out of thin air. As with most other scientific ‘breakthroughs’, it was the result of painstaking work refining, polishing and clarifying. Likewise, Fisher was not the only one thinking along such lines. Francis Edgeworth had developed similar ideas and described them in a paper published in 1908 [2], although this was not recognized until sometime after Fisher's paper appeared. Moreover, Fisher did not stop thinking about such issues with the publication of the 1922 paper, but continued to explore and develop the ideas further (e.g. [3,4]), and indeed throughout his life as he wrestled with the challenges which his ideas had thrown up.

The context in which the 1922 paper appeared was a confusing one. Least-squares methods were well understood for curve fitting, and Pearson had described both the *χ*^2^-test and the method of moments. Bayes theorem, the normal distribution and Student's *t* were all known. But this is a list of unconnected ideas, lacking a unifying infrastructure. In her biography of Fisher, his daughter Joan Fisher Box says ‘It is difficult now to imagine the field of mathematical statistics as it existed in the first decades of the twentieth century. By modern standards the terms of discourse appeared crude and archaic and the discussion extremely confused. … The whole field was like an unexplored archeological site, its structure hardly perceptible above the accretions of rubble’ [5, p. 62].

Fisher's aim, as is evident from the title of the 1922 paper, was to provide some sort of unifying theory, and, to facilitate this, he identified three fundamental objectives:

— *specification*, by which he meant choosing the relevant family of distributions;
— *estimation*, deciding how to derive statistics which are estimates of the parameters of the (hypothetical) population; and
— *distribution*, determining the distribution of the statistics.

Incidentally, I cannot help but comment that the late Dennis Lindley remarked that providing a unifying theory was also his aim when, as a young man, he was appointed to a position at Cambridge [6]. Given that Fisher and Lindley had similar aims, it is interesting that they came up with two such complementary approaches. In a way, though, they both had the same strategy: as we will see Fisher objected to the notion of inverse probability, and sought to do away with it. Lindley and others resolved the challenges of inverse probability by redefining what was meant by ‘probability’ (hence, de Finetti's ‘probability does not exist’ [7, p. x]).

After giving a list of definitions, Fisher's 1922 paper begins by commenting on ‘the prolonged neglect into which the study of statistics, in its theoretical aspects, has fallen’. It says ‘the basic principles of this organ of science are still in a state of obscurity’ [1, p. 310]. He attributes this sorry state of affairs to two considerations.

The first is the belief that if the subject matter is susceptible to uncertainties (‘greater or smaller errors’ [1, p. 311]) then precise definition of ideas or concepts is either impossible or unnecessary. Of course, with the benefit of the hindsight accumulated from observation of the immense advances that statistical tools have led to, one might nowadays state that if the subject matter is susceptible to uncertainties then the demand for statistical methods is all the greater. Although Rutherford had an element of truth in his famous observation that ‘if your experiment needs statistics, you ought to have done a better experiment’, he missed the fact that any investigation at the boundaries of knowledge must, almost by definition, have uncertainties and errors in measurement—and hence needs statistics to tease out the answers.

Fisher secondly attributed the dire state of early twentieth century statistics to the confusion arising from using the same word for the unknown true value of a parameter and its estimate. Indeed, although the word ‘parameter’ was occasionally used in physics at the time, it was virtually unknown in statistics. It was Fisher, explicitly using the terms ‘parameter’ and ‘statistic’ to distinguish between the usages in his 1922 paper, who introduced the terms and the distinction. (According to Stigler [8], using it 57 times in the paper—I made it 58.)

From Fisher's perspective, the key point about this second confusion is that it ‘would appear to suggest that the former quantity, and not merely the latter, is subject to error’. This perspective is fundamental to Fisher's view, since it allows him to go on to say that it is this which, in his opinion, ‘has led to the survival to the present day of the fundamental paradox of inverse probability, which like an impenetrable jungle arrests progress towards precision of statistical concepts’ [1, p. 311].

‘Direct probability’ refers to the chance aspects of making an observation. If we toss a fair coin, the (direct) probability of observing a head is, by definition, 1/2. If the coin is not fair (perhaps it is bent, for example), then the probability of getting a head may not be 1/2. Perhaps it is a little more, so that if we were to toss the coin a great many times we would expect to see rather more heads than tails. This probability—1/2 for a fair coin, but perhaps something different for a biased coin—is a parameter. The proportion of heads in a sequence of tosses is a statistic.

‘Inverse probability’ refers to the values that the parameter might take. We can imagine a distribution of possible values for the probability that the coin will come up heads—perhaps peaked at 1/2, this being what we think is the most likely value, but decaying as we move away from 1/2, to reflect the amount to which we think the coin might be biased. As Fisher said, conjecturing such a distribution for the value of the parameter suggests that the parameter and not merely the statistic is ‘subject to error’.

Looking back at Fisher's work, one can see the gradual development of his ideas—in particular in clarifying the notions of likelihood and maximum likelihood. The progression is matched with a gradually changing terminology; for example, from the ‘absolute criterion’, through the ‘optimum’, to ‘maximum likelihood’ in the 1922 paper.

The development is evident right from his first paper, written in 1912 while he was still an undergraduate student. Here he points out that neither of the two methods of estimation which were in use were without their problems. In least-squares curve fitting, one can get a different solution if the abscissa is transformed, while in the method of moments the choice of which moments to use is made without justification. Fisher then presents a solution, saying ‘we may solve the real problem directly’ [9] and pointing out that *P*, the probability of obtaining the observed data with different values of the parameters, can be used to choose between those values. Unfortunately, he confusingly and misleadingly describes this by saying, ‘the most probable set of values for the [parameters] will make *P* a maximum’ [9]—though he does pull back from this in the concluding section: ‘*P* is a relative probability only, suitable to compare point with point, but incapable of being interpreted as a probability distribution over a region’ [9]. In this paper, he seems to be slowly clarifying in his mind the distinction between *P* seen as a function of the data and *P* seen as a function of the parameters, a clarification which was complete by 1922.

For example, in his 1922 paper he corrects his error and introduces a new term to clarify things: ‘I must indeed plead guilty in my original statement of the Method of the Maximum Likelihood to having based my argument upon the principle of inverse probability … Upon consideration, therefore, I perceive that the word probability is wrongly used in such a connection: probability is a ratio of frequencies, and about the frequencies of such values we can know nothing whatever. We must return to the actual fact that one value of *p*, of the frequency of which we know nothing, would yield the observed result three times as frequently as would another value of *p* … I suggest that we may speak without confusion of the *likelihood* of one value of *p* being thrice the likelihood of another’ [1, p. 326].

Again, the gradual refinement of his ideas is apparent in his other papers. At the end of a paper published in 1921 [10], focusing on the distribution of the correlation coefficient, Fisher added a ‘Note on the confusion between Bayes rule and my method of the evaluation of the optimum’. He says, ‘My treatment of this problem differs radically from that of Bayes. Bayes [11] attempted to find … the actual *probability* that the population value lay in any given range. … Such a problem is indeterminate without knowing the statistical mechanism under which different values of *ρ* come into existence … What we can find from a sample is the *likelihood* of any particular value of *ρ*, if we define the likelihood as a quantity proportional to the probability that, from a population having that particular value of *ρ*, a sample having the observed value *r*, should be obtained’ [10, p. 24]. And again he stresses the difference in Fisher [1, p. 326]: ‘likelihood is not here used loosely as a synonym of probability, but simply to express the relative frequencies with which such values of the hypothetical quantity *p* would in fact yield the observed sample’.

He goes on to characterize the distinction between the two concepts: ‘Formally, therefore, [likelihood] resembles the calculation of the mode of an inverse frequency distribution. This resemblance is quite superficial: if the scale of measurement of the hypothetical quantity be altered, the mode must change its position, and can be brought to have any value, by an appropriate change of scale; but the optimum, as the position of maximum likelihood may be called, is entirely unchanged by any such transformation’ [1, p. 327]. This is an elaboration of the point he made about scale transformation in Fisher [9]. He stresses that likelihood ‘is not a differential element, and is incapable of being integrated: it is assigned to a particular point of the range of variation, not to a particular element of it’ [1, p. 327].

He also points out a further distinction between ‘this method and that of Bayes’, namely that (in estimating the proportion *p* of a population which are ‘successes’) Bayes used a uniform prior. After commenting on the fact that this produces ‘a vitally important piece of knowledge, that of the exact form of the distribution of *p*, out of an assumption of complete ignorance’ [1, p. 325], Fisher points out that this implies a non-uniform distribution for transformations of *p*, which might equally legitimately be chosen to parametrize the problem.

The originality and breadth of impact of the 1922 paper are demonstrated by the other concepts Fisher also described there. They include the notions of the *hypothetical infinite population* (the probability that an object takes a particular value is explicitly defined as the proportion of objects within a hypothetical infinite population which possess that value of the attribute), *consistency* (that, when applied to the whole population, the derived statistic should equal the parameter), *efficiency* (‘in large samples, when the distributions of the statistics tend to normality, that statistic is to be chosen which has the least probable error’ [1, p. 316]) and *sufficiency* (an estimator is sufficient if it contains the whole of the information about the unknown parameter that is contained in the sample). By virtue of Fisher's definitions of efficiency and sufficiency, a concept of *information* becomes relevant to statistical analysis. That a notion of information should emerge from his deliberations is not surprising: according to him, the aim of statistical methods is to reduce a body of data to a few quantities relevant to the objective in hand in such a way as to preserve all relevant information and exclude the irrelevant information.

Fisher, like all the best statisticians, was motivated by practical problems. He wanted to develop statistical methods which could handle the sort of data and the kinds of questions he encountered in real life, rather than promoting a mathematical idealization. And we find that, although this paper is ‘on the mathematical foundations’, it includes discussion of real examples. The way he used real practical problems is illustrated, on a small scale, by the way he corrected an assertion made by the astronomer Sir Arthur Eddington.

In parallel with his work leading towards likelihood, Fisher was exploring the notion of (what we now think of as characterizing the frequentist school of statistics) evaluating estimators using their sampling properties, such as the consistency and efficiency mentioned above. Eddington claimed that the best estimator of the standard error of a normal distribution was the mean of the absolute deviations. Fisher showed [12] that this was not the case, and in fact that the mean of the squared deviations contained ‘the whole of the information respecting’ the standard error. As he said in Fisher [1, p. 315]: ‘the discussions of theoretical statistics may be regarded as alternating between problems of estimation and problems of distribution. In the first place a method of calculating one of the population parameters is devised from common-sense considerations: we next require to know its probable error, and therefore an approximate solution of the distribution, in samples, of the statistic is calculated. It may then become apparent that other statistics may be used as estimates of the same parameter. When the probable errors of these statistics are compared, it is usually found that, in large samples, one particular method of calculation gives a result less subject to random errors that those given by other methods of calculation’.

If the 1922 paper brought the two strands of Fisher's work together, it was not quite as smooth a merger as he had initially hoped. The abstract for his presentation of the paper to the Royal Society in November 1921 (*Nature*, 24 November 1921) read ‘statistics obtained by the method of maximum likelihood are always sufficient statistics’. However, by the time the paper actually appeared in print, in 1922, this certainty had evaporated: ‘we require a method which for each particular problem will lead us automatically to the statistic by which the criterion of sufficiency is satisfied. Such a method is, I believe, provided by the Method of Maximum Likelihood, although I am not satisfied as to the mathematical rigour of any proof which I can put forward to that effect’ [1, p. 323].

Fisher was immensely creative, so perhaps it is not surprising not all of his ideas are mutually consistent. Recall John Maynard Keynes, when accused of being inconsistent, remarking that when the facts changed he changed his mind. Of Fisher, Efron has said: ‘Fisher usually wrote as if he had a complete logic of statistical inference in hand, but that didn't stop him from changing his system when he thought up another landmark idea’ [13, §4.3].

The 1922 paper is a long one, some 60 pages, and not all of it is of great interest to modern eyes. The second half of the paper is concerned mostly with Pearsonian families of curves, the method of moments and discrete distributions, and has had less lasting impact.

Fisher's paper of 1922 provided elements for a solid framework for statistical inference, and these elements are still central to practical applications. However, ideas develop, and it should not be seen as surprising that the framework Fisher sought to develop nearly a century ago has been elaborated, criticized and indeed challenged over time. Scientific progress is often a matter of identifying the cracks in concepts which manifest themselves as ambiguities and confusions, and then shining a searchlight into those cracks. This is very much the case with the discipline of statistics.

Fisher, in his forceful denunciation of inverse probability, marshalled several eminent authorities in support (‘The criticisms of Boole, Venn, and Chrystal have done something towards banishing the method … though we may agree wholly with Chrystal that inverse probability is a mistake … ’ [1, p. 311]), and described a process of gradually increased understanding of the inadequacies of the concept. However, Zabell [14] has pointed out that this portrait ‘does not appear entirely plausible’. Other eminent statisticians certainly supported the concept, and methods based on it were widely taught. Moreover, ‘virtually every textbook in probability written in English during the period 1886–1930 includes the topic, as well as most texts in French and German’ [14, p. 253]. Nonetheless, Fisher, along with Jerzy Neyman working in a closely related inferential paradigm, succeeded in delaying the development of methods based on notions of inverse probability for a quarter of a century. Zabell [14, p. 247] describes it as a ‘nearly lethal blow to Bayesian statistics’.

Of course, as any modern statistician knows, the blow was not *entirely* lethal. Rather than dismissing the notion of inverse probability, several thinkers, including Savage, de Finetti, Ramsey, Jaynes, Richard Cox, Jeffreys and Lindley, sought an alternative way of looking at it—very much a paradigm shift (or perhaps a paradigm resurgence, since this was the sort of perspective Bayes and others had had on probability). This alternative perspective has led to the so-called *Bayesian* school of statistics. The core of the development was the proof that any internally coherent approach to inference would have to be based on updating probabilities through Bayes theorem. (A system of probabilities is ‘incoherent’ if (for example) it is possible to find a combination of bets, each of which you would happily take, but which overall would guarantee a loss.)

The phrase Bayesian statistics is rather unfortunate, since *all* statisticians acknowledge the importance of Bayes theorem—a purely mathematical theorem of probability. The term is also rather unfortunate because it spans a wide range of rather different approaches to statistical inference, which may draw different conclusions when applied to practical problems. Nonetheless, they have the broad common basis that they relax the concept of probability from the relative frequency notion that Fisher espoused. In the Bayesian approach, any unknown quantity—like the parameter giving the probability that a coin will come up heads—is a random variable.

While difficulties remain in interpreting some of Fisher's concepts (not least his notion of *fiducial inference*), no inferential school is free of problems, conceptual or practical. With Bayesian approaches, for example, the interpretation of prior distributions as initial degrees of belief is all very well, provided genuine effort is made to determine what those are. All too often, however, priors are chosen on grounds of mathematical convenience, rather undercutting the conceptual basis. When we study logic, we learn that a valid logical argument can lead to incorrect conclusions if the premises are wrong. In an analogous way, a coherent argument based on incorrect premises is of limited value. We statisticians must always remember that our aim is to draw conclusions about the real world, based on models. Our models are not the reality. One must be wary of letting the tail of mathematical coherence wag the dog of the scientific question.

Cox [15, p. 197] says ‘frequentist analyses are based on a simple and powerful unifying principle. The implications of data are examined using measuring techniques such as confidence limits and significance tests calibrated, as are other measuring instruments, indirectly by the hypothetical consequences of their repeated use’. Clearly that makes very good sense: one ought to be uneasy about adopting an analytical tool which predominantly gave poor answers. With that as context, it is hardly surprising that domains such as those of regulatory authorities and the financial world make such heavy use of frequentist methods, based, in large measure, on Fisher's ideas, as described in his 1922 paper.

On the other hand, one might also remark that Bayesian analyses are likewise based on a simple and powerful unifying principle: that probability is not a property of the external world, but rather represents an internal degree of belief (about the possible values that particular parameters can take, for example).

Indeed, why stop there? One might add that the school of inference which adopts likelihood as the key inferential concept is also based on a simple sound unifying notion—Fisher's concept of likelihood, as described in Fisher [1].

The second half of the twentieth century saw a dramatic resurgence of interest in alternatives to the inferential approaches developed by Fisher (and the related approach developed by Neyman). Occasionally exchanges took place which were better suited to political debates than scientific discussions. Now, some years into the twenty-first century, the debate has matured. As I remarked above, the modern perspective is that a model is just a model, not the reality (‘All models are wrong, but some are useful’ [16]). Many alternative models may do just as effective a job—without any of them being ‘right’ or ‘wrong’. The same sort of conclusion applies to schools of inference. A good statistician, of whatever school, should be able to draw sensible conclusions. (With the corollary that a bad statistician, of whatever school, …) More generally, as Bayarri & Berger put it, ‘statisticians should readily use both Bayesian and frequentist ideas’ [17, p. 58].

Fisher's ideas have played an immense role in creating the modern world. Statistical ideas and inferences crop up everywhere, including medical, health and pharmaceutical research, manufacturing and quality control, all sciences from physics (the discovery of the Higgs boson was in large part a statistical challenge) through to psychology and sociology, economics and government, education, and agriculture and food production. No aspect of modern life remains immune from the application of statistical tools. Moreover, despite the growing interest in other inferential strategies, the overwhelmingly largest part of that impact is due to ideas originally formulated by Fisher.

There is sometimes a tendency to look at where we are now, look back at how we got here, and to see ourselves as at the culmination of a process of development. As far as statistical ideas and methods go, this would be a mistake: the evolution is continuing.

In large part, the evolution of statistics has been driven by new challenges, as its ideas spread into new domains. This can be seen from the development of experimental design for agriculture (an area which in a very real sense Fisher originated, and which subsequently spread into manufacturing, medicine and other areas), factor analysis and latent variable models in psychology (later being adopted in geology, finance, etc.), survival analysis being developed in medicine (and then being applied in a host of other areas) and so on. More recently hybrid Bayesian frequentist notions are being increasingly widely used, such as ensemble models, empirical Bayes methods and false discovery rate ideas.

Prior to Fisher, statistics was largely concerned with ‘matters of the state’. That meant large datasets and large-scale summaries of ‘vital statistics’, concerned with such things as birth and death rates. Despite his interest in asymptotic justifications, Fisher showed how the ideas could also be used in small sample problems, and this was a key influence on leading to the all-pervasive impact of statistical ideas and tools. But the world is moving on. We have entered a second era of ‘big data’. With electronic data capture technology, massive datasets are automatically accumulating—and with massive data comes massive opportunities, for discovery and for answering questions which we could previously not answer. Having said that, a cautionary note is appropriate: automatic data collection does not mean automatic answers to questions. Big data bring big challenges—perhaps even big enough for us to require new inferential ideas.

Automatic data capture also has another face, and one which promises to change the nature of statistics. Throughout the twentieth century, statistical practice consisted of collecting and analysing data, to answer specific questions (or, perhaps, to explore data to generate new questions). The modern world has opened a new front for statistics: automated analysis. Increasingly statistical inferential tools are embedded in machines around us: think of aircraft, of driverless cars, of route-finding software, of the intelligence embedded in mobile phone apps and so on. Some of this is Bayesian, but much is not: a great deal of it makes direct use of Fisher's ideas.

Fisher's 1922 paper shows a genius in full creative flow. But it shows this warts and all. It shows mistakes, backtracking, uncertainty. And it also shows how ideas build on others. It also perhaps shows a determination to stick to one's guns despite attacks and criticisms. Whether that is good is judged by the referee of history. Writing in 1950, Fisher said ‘I am still too often confronted by problems, even in my own research, to which I cannot confidently offer a solution, ever to be tempted to imply that finality has been reached (or to take very seriously this claim when made by others!)’ [18].

Bradley Efron summarized Fisher's contribution thus: ‘Let me say finally that Fisher was a genius of the first rank, who has a solid claim to being the most important applied mathematician of the 20th century. His work has a unique quality of daring mathematical synthesis combined with the utmost practicality. The stamp of his work is very much upon our field and shows no sign of fading. It is the stamp of a great thinker, and statistics—and science in general—is much in his debt’ [13, p. 113].

## Author profile

**David Hand** is Senior Research Investigator and Emeritus Professor of Mathematics at Imperial College London, where he was previously Professor of Statistics. He has research interests in classification, data mining, the foundations of statistics and applications of statistics, both for inference and for describing the state of the nation. He has published 28 books, most recently *The Improbability Principle: Why Coincidences, Miracles, and Rare Events Happen Every Day*. During his formative years as a statistician, he was witness to the sometimes heated debate between proponents of competing schools of inference, particularly the Bayesian and frequentist schools.
